# Across-language masculinity of oceans and femininity of guitars: Exploring grammatical gender universalities

**DOI:** 10.3389/fpsyg.2022.1009966

**Published:** 2022-11-24

**Authors:** Elena Dubenko

**Affiliations:** Institute of Philology, Taras Shevchenko National University of Kyiv, Kyiv, Ukraine

**Keywords:** grammatical gender, cognition, personification, grammatical gender universalities, semantic motivation

## Abstract

This is the first cross-language study to reveal nouns with invariable masculine or feminine grammatical gender assignments in nine gendered languages from different groups of one linguistic family. It evidences that many cases of gender universality have semantic motivation-an entity’s grammatical gender correlates with either traditional masculine/feminine connotations, or cultural and symbolic implications. The study’s findings also testify thematic preferences: most masculine grammatical gender universalities are found for the nouns denoting artifacts, whereas most feminine universalities are identified for abstract concepts. The apparent existence of grammatical gender universalities has a cognitive significance. From a psycholinguistic perspective, grammatical gender is viewed as a built-in personification pattern for speakers’ mental representations. This research presents cross-linguistic constants in conceptualizing the natural kinds, artifacts, and abstract concepts denoted by the considered nouns, as “male” or “female”.

## Introduction

The relationship between grammatical gender and thinking has been explored extensively in recent years. Psycholinguistic studies of grammatical gender and cognition prove that this grammatical category can shape habitual ways of perceiving the world in different language communities. The impact of grammatical gender on perception has been revealed for two-gender languages, such as Spanish, French, and Italian (Sera et al., 2002; Cubelli et al., 2011; Haertlé, 2017), and for three-gender languages, such as German, Greek, Polish, and Norwegian (Phillips and Boroditsky, 2003; Saalbach et al., 2012; Imai et al., 2014; Beller et al., 2015; Bender et al., 2016; Maciuszek et al., 2019; Pavlidou and Alvanoudi, 2019). Segel and Boroditsky (2011) found that grammatical gender provides a template for 78% of all personifications in visual arts. The impact of grammatical gender on thinking is also supported by many studies of bilinguals (Bassetti and Nicoladis, 2016). However, research results do not always converge: some scholars have found no relationship between grammatical gender and cognition (for instance, the third experiment in Ramos and Roberson, 2010), while others evidence the effects of grammatical gender only for languages with a two-gender system (Sera et al., 2002), or for the nouns denoting animals and not artifacts (Vigliocco et al., 2005). According to a recent systematic survey of 43 pieces of experimental research on grammatical gender and linguistic relativity, such divergence of findings reflects that the influence of grammatical gender on concepts is “strongly task- and context-dependent” (Samuel et al., 2019, p. 1784). This explanation likely accounts for the apparent conflict between failings to replicate the widely cited results obtained by Boroditsky et al. (2003) (Elpers et al., 2022), and the contemporaneous studies completing Boroditsky’s findings (Williams et al., 2021; Mecit et al., 2022); the latter attest that grammatical gender does influence how individuals mentally represent objects of reality, thus shaping anthropomorphism tendencies. The field’s state-of -the-art, undoubtedly, indicates the necessity for more profound investigation.

Meanwhile, despite all these disparities, experimental studies testify that native speakers of a gendered language have low awareness of grammatical gender arbitrariness, perceiving a noun’s grammatical gender as consistent with the gender characteristics of the denoted object. In other words, they consider that grammatical gender is semantically motivated (Bassetti, 2014).

However, prior investigations of grammatical gender identification of objects and phenomena in different languages have not been sufficiently systemic. Typology and cognition were long treated as basically different domains, and scholars have only recently started to discuss exciting perspectives of cooperation between typology and cognitive linguistics (Croft, 2016; Corbett and Fedden, 2018). Another issue is the tangible lack of cross-language studies seeking to uncover in grammatical gender patterns those ethnospecific regularities that are immediately related to world view aspects. Typologically, there are two possible angles for such investigation: searching for *discriminants* or *commonalities* in different grammatical gender systems.

In psycholinguistic studies exploring the effects of grammatical gender on speakers of various languages, the main focus is language-induced *differences* in the mental representations of respondents belonging to different cultural groups. For example, investigating the effect of grammatical gender on object categorization, Cubelli et al. (2011) report that semantic processing in Spanish, Italian, and English is affected by lexical-grammatical properties of each particular language. These findings, and the results of other cross-linguistic studies concerning the impact of grammatical gender on perception (Kousta et al., 2008; Bender et al., 2011, 2016) have relevance for the hypothesis of linguistic relativity. At the same time, modern scholarly contributions on the topic disregard grammatical gender commonalities.

It is in line with some overall tendencies in modern linguistics, particularly, the view that linguistic diversity should be the crucial subject of cognitive science because language universals are so few and inconsiderable that may be regarded as mythical (Evans and Levinson, 2009).

Both absolute and statistical universalities are viewed as working hypotheses-theoretical speculations, not reality (Bickel, 2014). Proponents of this standpoint see language as a bio-cultural phenomenon representing invariant mental mechanisms and variable cultural traditions. Therefore, it is proposed to abandon universal assumptions in favor of Boasnian “methodological relativism,” which presupposes that language is first analyzed in its own terms and then compared to other languages (Levinson and Evans, 2014). Catania (2009) suggests extending this two-track model of biological and cultural evolution to a three-track one incorporating an individual’s language repertoires in ontogeny. At the same time, supporters of less radical ideas as for linguistic diversity emphasize that Evans and Levinson’s approach is based principally on analyzing Chomskyan Universal Grammar, while neglecting other universalist aspects. For instance, Evans and Levinson examine mostly substantive variation of language and pay no due attention to formal universals (Nevins, 2009); they also ignore common processes at the expense of differences between languages at a superficial level (Tallerman, 2009). Since there is no distributional evidence for the postulates of Universal Grammar, Cristofaro (2012) suggests that scholars cease further searches for innate language properties and typological universals. Another key consideration is that although language universals give revealing insights into language structure, they need a structural, historical, and functional explanation (Moravcsik, 2012). It is also claimed that Evans and Levinson exaggerate linguistic diversity while a one-sided presentation of the issue cannot be regarded as satisfactory in the context of cognitive tasks set by contemporary linguistics [Pinker and Jackendoff, 2009; Rizzi, 2009; see also the related polemics of Deutscher (2010) and McWhorter (2014)]. In my opinion, the truth lies somewhere in the middle, and, neither a neo-Whorfian doctrine nor a universalist standpoint should be carried to its extreme, as a holistic approach is necessary for effective exploration of the cognitive dimensions of language. In discussing meaning-making in literary translation, Boase-Beier (2019) rightfully points out the inappropriateness of excessively foregrounding linguistic diversity, given the Sapir-Whorf hypothesis covers only part of its founders’ views: like Wilhelm von Humboldt, they considered the unique worldview encoded in each language coexists with certain universal facets of language.

Adopting a cross-linguistic perspective, this study aims to reveal the items that have the same grammatical gender assignments in various languages and hypothetically offer identical mental representation framework to their respective speakers. Another immediate purpose is to clarify whether universal tendencies in grammatical gender assignments are purely coincidental or explained by certain dimensions of their semantic motivation.

The notion of *universal* is considered rather challenging and controversial in contemporary typology, so its meaning for the purposes of this paper needs to be specifically defined. The study makes no claims that the presented identical gender patterns indicate a total cross-linguistic universality. Its more modest contribution is to offer evidence on the potential existence of grammatical gender universalities, which future research is expected to reinforce or refute based on a much larger scope of language material. Accordingly, I use the working term *universalities* to describe any *universal tendencies revealed by analyzed data from one language family.*

Although the research material is drawn predominately from languages of the Indo-European family, which are intrinsically related, this does not diminish the universality value of the study findings. Moreover, the revealed universal patterns should not be dismissed as expected, since the linguistic characteristics underlying the relatedness of Indo-European languages do not include grammatical gender. The latter were grouped together because “they share a number of items of basic vocabulary, including grammatical affixes, whose shapes in the different languages can be related to one another by statable phonetic rules”(Jasanoff, 2022). Meanwhile, the diversity of grammatical gender assignments is virtually notorious not only in a cross-linguistic respect, but even within the same language over time. For instance, grammatical gender assignments of nouns in Old English do not correlate with the gender ascribed to corresponding entities in Modern English (Dubenko, 2015: p. 102–103).

Likewise, similar grammatical gender patterns found in Hebrew cannot realistically be explained by some relatedness between Indo-European and Afro-Asiatic languages. Most scholars maintain that there are no genealogical links between Indo-European languages and the languages of other families (Kallio et al., 2018).

Finally, despite the Indo-European family comprises only 6% of all the world’s languages, it is the largest language family in terms of speakers, over 3 billion people, or 46% of the world’s population (Ethnologue; Wikipedia, 2022). This is important given the study’s focus on not only purely linguistic but also psychological aspects: the circumstance that nearly half the world speaks Indo-European languages is responsible for the distribution of hypothetical universal mental representations of the analyzed entities.

In summary, the scope of the analyzed material allows to initiate a discussion about grammatical gender universalities, which I see as the paper’s primary task.

## Materials and methods

To secure a tangible difference in grammatical gender patterns, nine languages were selected for a comparative analysis, presenting different language groups of the Indo-European family: the Germanic group (German), Greek group (Modern Greek), Italic group (French, Italian, Spanish), and all three branches of the Slavic group, the Western branch (Polish), Southern branch (Bulgarian), and Eastern branch (Russian, Ukrainian).

The universalities identified in this one linguistic family were subsequently compared with a similar corpus of items in Hebrew, which belongs to the Semitic group in the Afro-Asiatic family.

A total of 529 nouns were analyzed in this research. They were selected from the 5,000 most frequently used words according to the Corpus of Contemporary American English (Davies, 2015). This source was chosen for two reasons. First, frequency greatly determines an entity’s significance in the processes of world conceptualization, which enhances the probability of its personification by native speakers. Second, reliance on such a corpus allows to avoid the problems of researcher subjectivity.

However, it is important to note that frequency of use was not an end in itself. The Corpus of Contemporary American English served only to isolate the nouns that signify the major concepts of human physical reality and spiritual life which can be potentially personified. Although the compared languages differ, their speakers inevitably share certain universal entities of material and spiritual culture. Thematically, the analyzed words belong to domains usually considered in psycholinguistic studies of grammatical gender and cognition. They denote artifacts, natural objects and phenomena, and abstract concepts.

More specifically, the words include foundational entities of human existence, such as artificial objects encountered in everyday life (e.g., architectural constructions, furniture pieces, household utensils, transport vehicles, technological gadgets of everyday use), natural kinds (e.g., seasons, celestial bodies, weather conditions, elements of geographical relief, the commonest entities in organic and inorganic world), and abstract concepts of basic physical, mental and psychic states; positive and negative emotions; key notions of intellectual knowledge, morality, law, religion, aesthetics, and language.

The compiled list of words was filtered for personifiable items, thereby excluding lexical units with no obvious masculine or feminine connotations: examples include anatomical terms (*limb, hand, foot*), clothing details (*collar, pocket*), and abstract nouns of poor personification value (*statement, role, therapy, shift*). Because the pool lacked some entities traditionally depicted as human personifications in literature, especially in poetic works, it was complemented with 14 such nouns: *gratitude, solitude, revenge, jealousy, hostility, envy, eternity, faith, paradise, cradle, thunder, drought, frost, volcano.*

The grammatical gender of the nouns under consideration was identified by consulting the following dictionaries: *AΓΓ*Λ*O–E*ΛΛ*HNIKO*Λ*EΞKO (2004); Complete English-Russian Russian-English Dictionary* (Muller, 2013)*; Dictionary of modern Hebrew. Russian-Hebrew. Hebrew-Russian* (Podolskiy et al., 1993)*; Dictionnaire Russe-Français* (Scerba and Matoussevitch, 1993); *Dizionario Russo Italiano Italiano Russo* (Kovalev, 1999); *Gran Diccinario Ruso-Español* (Turover and Nogueira, 2000); *Neues Deutsch-Russisches Russisch-Deutsches Wörterbuch* (Baikow and Böhme, 2009); *Russian-Bulgarian dictionary* (Chukalov, 1986); *Russian-Hebrew Universal Dictionary* (Kharakh, 1995); *Słownik polsko-rosyjski rosyjsko-polski* (Kowalowa, 2010); *Ukrainian-Modern Greek Dictionary* (Klymenko et al., 2008).

All of them are academic editions compiled on the basis of a representative corpus of multi-genre lexicographic sources, with the direct participation of native speakers.

## Results

The investigation revealed three groups of nouns that are relevant for the discussion of grammatical gender universality in a cross-linguistic perspective.

The first group can be called ***absolute (or exceptionless) grammatical gender universalities*** as these nouns have identical grammatical gender in all analyzed Indo-European languages. Among them are 58 ***non-alternative absolute grammatical gender universalities***, with only a masculine or feminine variant of gender assignment in all compared languages. These comprise 13 masculine grammatical gender universalities (*ocean, volcano, organism, stadium, month, corridor, rhythm, triumph, wind, balcony, success, sleep, sense)* and 45 feminine grammatical gender universalities (*atmosphere, drought, clinic, library, guitar, bomb, reality, independence, democracy, fashion, career, virtue, friendship, responsibility, discipline, emotion, morals, music, comedy, tragedy, weakness, criticism, hostility, religion, harmony, nature, beauty, grace, eternity, street, kitchen, night, rose, rage, threat, wound, bottle, irony, freedom, energy, idea, poetry, aid, offense, melody*) (see Supplementary appendix A).

The remaining absolute grammatical gender universalities allow some minor gender alternatives. Such grammatical gender variations of the entities under consideration have a very limited character, and are typically found in only one or two of the nine compared languages. For example, in Italian the concept *soul* is denoted by two lexemes of different grammatical gender: *ànima* (feminine), and *animo* (masculine); likewise in Spanish the noun *computer* has masculine (*computador*) and feminine (*computadora*) alternatives. The ***absolute grammatical gender universalities of alternative type*** include 13 masculine universalities (*stream, hill, park, diamond, temple, port, motor, computer, character, style, myth, terror*, *hurricane*) and 18 feminine grammatical gender universalities (*plain, cave, moisture, midnight, lamp, joy, soul, hope, strength, advertising, truth, wisdom, defense, disease, slander, culture, revenge, gratitude*). In Supplementary appendix B alternative variants are set in bold.

By analogy with Evans and Levinson’s (2009, p. 437) logical types of universal statements, the second group of words (29 masculine and 16 feminine gender nouns) may be treated as ***conditional* (*or restricted*) *grammatical gender universalities.*** These lexical units show the same gender identification in eight out of the nine Indo-European languages under consideration (see Tables 1, 2).

**TABLE 1:** Restricted masculine grammatical gender universalities.

Nouns with dominant masculine grammatical gender identification	Contrasting gender pattern	
	Gender	Language
Palace, van, bus, helicopter, tank, telephone, laser, carpet, pencil, sweater, oxygen, gene, nerve, crystal, end, humor, rice, apparatus	Neuter	Greek
Rice, apparatus, TV set, tap/faucet, day, thunder	Feminine	Greek
Wood, garden, trunk	Feminine	Bulgarian
Airport	Neuter	Polish
Satellite	Feminine	Polish
World	Feminine	German
Paradise	Neuter	German

**TABLE 2:** Restricted feminine grammatical gender universalities.

Nouns with dominant feminine grammatical gender identification	Contrasting gender pattern	
	Gender	Language
Drop, skirt, faith, glory, envy	Masculine	German
Land, prayer	Neuter	German
Galaxy	Masculine	Greek
Earth, passion, tear	Neuter	Greek
Humanity, anxiety	Neuter	Bulgarian
Guilt, cradle	Masculine	French
Fatigue	Neuter	Polish

A complete list of the identified restricted grammatical gender universalities is given in Supplementary appendix C (with variants that deviate from the dominant tendency set in bold). Among the absolute grammatical gender universalities, feminine universalities are predominant (77.9% compared to 22.1% for masculine universalities). However, this pattern is less evident for the alternative, insertion, grammatical gender universalities (58.06% vs. 41.94%), and does not hold for the restricted universalities (35.56% vs. 64.44%). Although statistical analysis of three groups indicates the dominance of feminine grammatical gender universalities [70.79%; χ^2^ (1, *N* = 134) = 15.33; *p* < 0.001; odds ratio = 4.39], this prevalence is more or less counterbalanced by the cluster of nouns that can be called ***non-feminine grammatical gender universalities*** (see Supplementary appendix D). These words have masculine grammatical gender identification in two-gender languages, and either masculine, or neuter gender identification in three-gender languages (*theater, museum, cemetery, aircraft, radar, instrument, cabinet, knife, piano, drum, diary, document, coat, talent, feeling, miracle, evil, sky, climate, earthquake, mineral, gold, iron, marble, baby, afternoon, grain, casino, right, crime, costume*).

Semantically, a substantial proportion of masculine grammatical gender universalities, both absolute and relative, are nouns denoting artifact objects (41.8% compared to 30.9% for natural kinds and 27.3% for abstract concepts), This tendency is more evident for non-insertion, grammatical gender universalities (53.6% compared to 32.1% for natural kinds and 14.3% for abstract concepts). Meanwhile, insertion, grammatical gender universalities show contrasting semantic preferences, with an overwhelming prevalence of nouns signifying abstract concepts (71.8% compared to 15.4% for natural kinds and 12.8% for artifacts). In statistical terms, 68.75% of masculine and non-insertion, grammatical gender universalities represent artifacts, while 80% of feminine universalities denote abstract concepts, χ^2^ (1, *N* = 107) = 23.56, *p* < 0.001; odds ratio = 8,8.

A considerable proportion of the objects and phenomena denoted by the above-listed gender universalities can be easily linked to features traditionally associated with the masculine and feminine: ***activity, strength, aggressiveness, hardness*,** and ***big size***; for masculinity, ***beauty, grace*,** and ***small size***–for femininity.

The adherence to masculine characteristics appears to be evident in masculine and non-feminine grammatical gender universalities.

The feature ***activity*** is inherent in transport vehicles and some other items presupposing the idea of movement: *van, bus, tank, aircraft, helicopter, satellite, motor, radar, laser, wind, stream.* Logically, active entities are also represented by artifacts which perform human-mediated actions or serve as means of information transmission: *apparatus, instrument, computer, telephone, TV set.* The semantic dominant of ***strength*** or potency is transparent in such notions as *ocean, thunder, volcano, earthquake, hurricane, triumph*, and *shock*. Some of them can also be associated with ***aggressiveness***, which appears quite conspicuous in the entities *knife, evil*, and *terror*. Another set of masculine and non-feminine grammatical gender universalities share the semantic characteristic of ***hardness*** (*diamond, iron, mineral, crystal, marble, gold*). Moreover, the semantic feature of ***big size*** is clearly present in such notions as *world, sky, ocean, wood, stadium, temple, palace, theater, aircraft, helicopter.*

In a similar way, a handful of feminine grammatical gender universalities appear to correlate with feminine characteristics. The semantics of ***beauty*** and ***grace*** are directly represented by those very words *beauty*, and also by notions with an aesthetic semantic dominant, such as *harmony*, *poetry*, and *music*. The lexeme *drop* semantically suggests ***small size***. There are also four items that have traditional associations with females (*cradle*, *kitchen, skirt, fashion*), while the noun *guitar* designates an object whose form is suggestive of a female figure.

It is also possible to trace a symbolic dimension of semantic motivation in some feminine grammatical gender universalities. Symbolically, the archetypical image of woman is delineated in three main guises: the superior aspect (Sophia or Mary), the Magna Mater aspect, and the inferior aspect (Eve or Helen) (Cirlot, 2001). The first aspect correlates with the anima of Jungian psychology, a personification of the moral, intellectual, and emotional in their supreme form. It corresponds to the entities: *morals, virtue, responsibility, discipline, religion, faith, wisdom, truth, idea, culture, soul, humanity, gratitude, hope, joy, friendship, aid, defense*. The second aspect, which implies the concept of motherland or Mother-Nature, is apparent in the items *nature, earth, land*. The third aspect presents the inferior emotional and instinctive side of woman: *emotion*, *passion, rage, envy, revenge*, *hostility.* Analogically, the semantic motivation for the feminine grammatical gender universalities *night, midnight* may be found in the classical symbolic correlation between night and the feminine, the unconscious (Cirlot, ibidem). Likewise, a connection can be traced between the feminine grammatical gender of *poetry* and mythological value of the concept. According to classical mythology, poets and bards derive their power from the Muses, the inspiring goddesses of song and poetry who live in Olympus, “and sing festive songs at the repast of the immortals. They bring before the mind of the mortal poet the events which he has to relate, and confer upon him the gift of the song” (Smith, 1884, p. 529). Therefore, poets are often called the sons of the Muses or their disciples. Moreover, various kinds of poetry are represented by different female divinities: Calliope, the Muse of epic poetry, Euterpe, the Muse of lyric poetry, Erato, the Muse of erotic poetry, Thalia, the Muse of merry or idyllic poetry (Ibid., p. 530).

In this context, the gender identifications of *rhythm* (masculine) and *melody* (feminine), two absolute grammatical gender universalities, correspond to ancient philosophical concepts. In particular, they are in keeping with Quintilian’s idea that melody is female because it presents an inactive and formless matter, while rhythm that molds the melody and “moves it in a determinate order, playing the part of the maker in relation to the thing made” is male (Baker, 1989, p. 445).

Overall, gender connotation is more typical of masculine grammatical gender universalities (74.5%), while symbolic implications are observed predominately in feminine universalities (93.5%), χ^2^ (1, *N* = 77) = 35.98, *p* < 0.001; odds ratio = 46.14.

Finally, nouns representing absolute grammatical gender universalities in the nine Indo-European languages were compared with identical lexical units in Hebrew. Analysis revealed that 83.15% of the identified universalities have the same grammatical gender value in this Afro-Asiatic language. The only exceptions are the masculine Indo-European universalities *wind, balcony, success*, which have feminine gender assignments in Hebrew, and the feminine Indo-European universalities *nature, beauty, grace, eternity, street, kitchen, night, rose, rage, threat, wound, bottle*, which in Hebrew belong to the masculine gender.

Meanwhile, 87.1% of Indo-European non-feminine universalities are supported by the Hebrew data.

## Discussion

The study shows a cross-linguistic universality of grammatical gender assignments for a sizeable group of key entities representing abstract concepts, artifacts, and natural kinds. The absolute universalities identified from nine Indo-European languages, with parallels in Hebrew, should be considered within a broader than ten languages context. Nevertheless, this research provides evidence of universalist grammatical gender tendencies that testify to the existence of similar male and female conceptualization patterns in the analyzed languages.

Evans and Levinson (2009) dismiss the existence of language universals, contending that any strong tendencies of universality are explained by connections between languages under consideration (i.e., appurtenance to one language group). Relatedly, Everett (2012) argues that similar linguistic features can appear only if the compared languages belong to “the same language family, the same cultural heritage” (2012, p. 84). These scholars’ reasoning is not applicable to the languages analyzed in the study, which belong to not only different language groups but also different language families. It would also be rash to assume that absolute grammatical gender universals are explained by loanwords being automatically assigned the same gender in languages that import them. On the contrary, there is evidence that the borrowing of nouns into gendered languages is carried out with the dominance of *semantic* gender assignment constraints (Audring, 2008; Thornton, 2009). This conclusion seems especially important, since the exploration of gender assignment to loanwords and neologisms is dubbed as “a continuously running experiment, which allows us to verify the assignment system in the languages in question” (Corbett, 1991, p. 71). Studies of a similar, but not absolutely identical, process of bilingual code-switching yield variable results: their findings show consistency with semantics-mediated cross-linguistic influence (Nicoladis et al., 2021), but also suggest that code-switching is impacted by various linguistic and extra-linguistic factors (Bellamy and Parafita Couto, 2022). The authors of both studies emphasize the need for further extended exploration. Accordingly, the implications of the above-mentioned results for gender universality will become clear through an all-sided investigation of the issue with the later application of different task types to highlight various aspects of cognition.

The only assumption that can be made now concerns an operational concept of these studies such as masculine default strategy. Logically, the preference for the masculine default strategy in code-switching appears to imply the existence of masculine gender universalist tendencies. However, this is definitely not the case for most non-alternative absolute feminine gender universals identified in this study. These apparent loanwords retain their gender affiliation in all the analyzed languages {e.g., democracy [Bulgarian: демокрация (f); French: démocratie (f); German: Demokratie (f); Greek: δημοκρατία (f); Hebrew: (f) דֵמוֹקרָטִיָה; Italian: democrazia (f); Polish: demokracja (f); Russian: демократия (f); Spanish: democracia (f); Ukrainian: демократія (f)]}.

According to recent findings, the masculine default strategy in Spanish is immediately connected to asymmetries in the distribution of nouns between masculine and feminine genders (Beatty-Martinez and Dussias, 2019). Although any cross-language generalizations on this matter are necessarily tentative, the fact that languages differ in gender distribution may influence gender assignment. For instance, masculine and feminine gender are distributed approximately equally in Spanish (Bull, 1965), and the same is true for French (56% masculine vs. 44% feminine) (Roché, 1992); meanwhile, of the German nouns, 46% are feminine, 34% masculine, and 24% neuter (Duden, 2022). Some authors argue that the more salient position of the masculine gender in Romance languages increases anthropomorphism tendencies and leads to more robust grammatical gender effects in Spanish or French, as compared to German (Speed and Majid, 2019, p. 5).

However, this standpoint appears contentious in view of the evidence that such effects equally hold for other three-gendered languages (Bender et al., 2016; Pavlidou and Alvanoudi, 2019). Therefore, one cannot state definitely that the speakers of Romance languages differ from Greek or German speakers in their perception of masculine gender universalities.

This study’s results also challenge the assumption that the criteria for assigning grammatical gender to words vary across cultures, and cannot be explained logically. They reinforce the claim of some researchers that grammatical gender originates in the metaphorical extension of natural gender to inanimate objects and abstract concepts, or in personification. Such treatment of grammatical gender is observed in the works of ancient Greek scholars (Protagoras), in the notional view of this category that dominated from the late 18th to the late 19th century (Herder, Adelung, Humboldt, Grimm), and in the early 20th century theories influenced by sociological, psychological and anthropological doctrines (Meillet, Martinet, Sapir, Whorf) (see Kilarski, 2007). The application of notional criteria to grammatical gender is supported by seminal typological works, which testify that gender assignment systems in all gendered languages have a semantic basis (Aksenov, 1984; Corbett, 1991). The phenomenon of “metaphoric gender” (Baron, 1986) has been confirmed in a multiple studies of different languages: English (Pawley, 2004), German (Zubin and Köpcke, 1986), Dyirbal (Dixon, 1982).

Manifesting in the analyzed grammatical gender universalities are dimensions of semantic motivation that align with the traditional motivation bases in earlier linguistic and psycholinguistic studies of grammatical gender. First, these are masculine or feminine connotations of the entity (*activity, strength, aggressiveness, big size, hardness*–for masculine gender referents, and *beauty, small size*–for feminine gender referents). Oppositions such as *active* vs. *passive*, and *big size* vs. *small size*, respectively, signifying male and female characteristics, were also frequently applied by English grammarians of the 19th century. They postulated that objects and abstract notions implying such male features as *big size, power*, and *strength* become associated with masculine gender, whereas those perceived as *small, tender* and *weak* tend to develop a stable connection with feminine gender (Taylor, 1804). This kind of semantic motivation is repeatedly mentioned in the findings of psycholinguistic studies: for *size*, small has a feminine connotation (Bassetti, 2014), while *strength/aggressiveness* and *beauty*, respectively, correspond to male and female features (Millis, 1986; Zubin and Köpcke, 1986; Konishi, 1993; Bassetti, 2014).

This study’s results also support another kind of semantic motivation described in psycholinguistic research as cultural references or symbolic representations, as found in mythology, iconography, and fairy tales (Bassetti, 2014). The semantic motivation of grammatical gender in the analyzed universalities is rooted in the symbolic meaning attached to these entities even in non-gendered languages. For instance, according to prior research of the personification patterns in Anglo-American poetry from the 18th to the 20th century, the masculine grammatical gender universalities *ocean, wind* and *hurricane* are viewed as masculine entities while the insertion, grammatical gender universalities *soul, nature, music, liberty*, and *beauty* have female personifications (Dubenko, 2019).

The dimensions of semantic connotations delineated in this study are derived from analyzing grammatical gender universalities in only ten languages. They may be confirmed or contradicted for each particular entity in future research investigating other languages. For instance, some masculine gender universalities within the category *big size* (e.g., *wood*, *aircraft*) could be found to have feminine grammatical gender assignment in some other language, thus losing the status of masculine grammatical gender universalities and any connection with the *big size* criterion. However, in this context, I wish to emphasize Evans and Levinson’s contention that non-universal but *significant recurrent patterns* are “solutions satisfying multiple design constraints, reflecting both cultural-historical factors and the constraints of human cognition” (2009, p. 429). On this basis, recurrent language commonalities should be regarded as motivated or sensical solutions, rather than results of “pure chance and complete accidents of language contact” (Goldberg, 2009, p. 455).

The proportional prevalence of artifact items in the masculine grammatical gender universalities is consistent with psycholinguistic research results. A universal preference for masculinity while assigning gender to artifact items was revealed in experiments involving English speakers (Mullen, 1990; Sera et al., 1994; Forbes et al., 2008). However, the previously reported tendency to assign feminine features to natural kinds was not supported by the present study: both natural kinds and artifacts have equally low shares in the identified feminine grammatical gender universalities, whereas abstract concepts are obviously dominant. The frequency of abstract concepts among feminine universalities correlates with the exclusively female gender of personified abstractions denoting emotions, philosophical notions, virtues, and vices “in Hellenic, Roman, and much medieval art and allegorical literature” (Paxson, 1998, p. 149).

There are two main avenues for further research on grammatical gender universalities. The first one is to continue this study’s line of investigation by analyzing other gendered languages, producing evidence that either confirms or dismisses the idea of absolute grammatical gender universals. As the study shows universal grammatical gender tendencies in Indo-European languages, future research should involve languages from different families. Such exploration appears especially intriguing and promising given the extensive support found in Hebrew for Indo-European grammatical gender universalities. It would also be appropriate to supplement the pool of lexical units with other entities, which may show similar universality of grammatical gender assignments. Another key research avenue is to clarify whether the entities in the categories of both absolute and restricted grammatical gender universals receive a specific treatment in psycholinguistic experiments on grammatical gender and thinking. The present study aimed to identify grammatical gender universalities in the explored Indo-European languages through of a cross-linguistic analysis. A logical next step is to examine peculiarities of their cognitive processing by native speakers of these languages, who can assess the degree of masculinity or femininity of each absolute, restricted or non-feminine grammatical gender universality. It is crucial for such experiments to have heterogeneous task designs, thereby elucidating which particular tasks and contexts produce the results confirming or refuting the gender value of each universal in psycholinguistic terms. However, it is quite reasonable to primarily employ the task types used in those psycholinguistic studies that attest the greatest support for grammatical gender effects, being, evidently, most related to the aspects of cognition affected by grammatical gender. These are sex assignment (for instance, Pavlidou and Alvanoudi, 2019) and voice choice (for instance, Haertlé, 2017). According to a systematic review of psycholinguistic research on grammatical gender (Samuel et al., 2019), by task type parameter, sex assignment correlates to 66% of support, and 26% of mixed support for linguistic relativity, and voice choice to, correspondingly, 64% and 22%.

## Conclusion

To the author’s knowledge, this is the first cross-linguistic study to focus on grammatical gender universality. The research revealed multiple universalities across languages from one linguistic family. The findings can be summarized in three main conclusion.

First, through a comparative analysis of the grammatical gender identification of nouns in ten gendered languages, this study proved the existence of masculine and feminine grammatical gender universalities, some absolute and others restricted in character. The absolute grammatical gender universalities are found in all ten analyzed languages, while the restricted universalities show gender uniformity in nine out of the ten languages. As both two- and three-gendered languages were included, the study also identifies a group of non-feminine grammatical gender universalities–nouns with only masculine or neuter grammatical gender identification in all ten languages. In light of the gender congruency effect, these findings testify that hypothetically analogous ways of conceptualizing objects of reality as “male” or “female” are shared by speakers of the considered languages.

Second, the gender of a grammatical universality shows a certain dependence on the noun’s semantic category. The proportion of feminine grammatical gender universalities is tangibly higher among abstract concepts, while masculine grammatical gender universalities are more frequent among artifacts. From a cognitive perspective, this suggests that the items of the analyzed spheres tend to be cross-culturally perceived as predominately masculine or feminine entities, with identical grammatical gender matrices encoded in the examined languages.

Third, the results support a transparent semantic motivation of grammatical gender universalities, linked in many cases, to either male and female connotations, or some cultural and symbolic implications of archetypal character.

In closing, I wish to emphasize that this study’s primary aim is to bring grammatical gender universalities to the scholarly agenda, encouraging wide academic attention. This research needs to be extended to other gendered languages and the implications of its findings require further, deeper investigation.

## Data availability statement

The original contributions presented in this study are included in the article/Supplementary material, further inquiries can be directed to the corresponding author.

## Author contributions

ED wrote the manuscript, contributed to the article and approved the submitted version.
