# Corrigendum: Across-language masculinity of oceans and femininity of guitars: exploring grammatical gender universalities

**DOI:** 10.3389/fpsyg.2023.1233792

**Published:** 2023-07-13

**Authors:** Elena Dubenko

**Affiliations:** Institute of Philology, Taras Shevchenko National University of Kyiv, Kyiv, Ukraine

**Keywords:** grammatical gender, cognition, personification, grammatical gender universalities, semantic motivation

In the published article, the Funding statement was omitted in error. The correct Funding statement appears below.

