# Supplementary material for: Across-language masculinity of oceans and femininity of guitars: Exploring grammatical gender universalities
Source: Front Psychol. 2022 Nov 24;13:1009966. doi: 10.3389/fpsyg.2022.1009966 (PMC9731155; doi:10.3389/fpsyg.2022.1009966)
Supplement: Supplementary file 1 [file Data_Sheet_1.pdf]

APPENDIX A

Non-alternative absolute grammatical gender universalities

Table 1. Masculine gender universalities

ENGLISH	UKRAINIAN	RUSSIAN	POLISH	BULGARIAN	GERMAN
1. ocean	океан (m)	океан (m)	ocean (m)	океан (m)	Ozean (m)
2. volcano	вулкан (m)	вулкан (m)	wulcan (m)	вулкан (m)	Vulcan (m)
3. organism	організм (m)	организм (m)	organizm (m)	организъм (m)	Organismus (m)
4. stadium	стадіон (m)	стадион (m)	stadion (m)	стадион (m)	Stadion (m)
5. month	місяць (m)	месяц (m)	miesiąc (m)	месец (m)	Monat (m)
6. corridor	корідор (m)	коридор (m)	korytarz (m)	коридор (m)	Korridor (m) Gang (m)
7. rhythm	ритм (m)	ритм (m)	rytm (m)	ритъм (m)	Rhythmus (m)
8. triumph	тріумф (m)	триумф (m)	triumf (m)	триумф (m)	Triumph (m)
9. wind	вітер (m)	ветер (m)	wintr (m)	ветер (m)	Wind (m)
10. balcony veranda	балкон (m)	балкон (m)	balkon (m)	балкон (m)	Balkon (m)
11. success	успіх (m)	успех (m)	sukces (m)	успех (m)	Erfolg (m)
12. sleep	сон (m)	сон (m)	sen (m)	сън (m)	Schlaf (m) Traum (m)
13. sense	сенс (m)	смысл (m)	sens (m)	смисъл (m)	Sinn (m)

Table 1. (continuation)

FRENCH	SPANISH	ITALIAN	GREEK
1. océan (m)	océano (m)	oceano (m)	ωκεανός (m)
2. volcan (m)	volcan (m)	vulcano (m)	ήφαίστειο{ν} (m)
3.organism(m)	organismo (m)	organismo (m)	οργανισμός (m)
4. stade (m)	estadio (m)	stadio (m)	στάδθιο (m)
5. mois (m)	mes (m)	mese (m)	μήνας (m)
6. corridor (m) couloir (m)	corredor (m) pasillo (m)	corridoio (m)	διάδρομος (m)
7. rythme (m)	ritmo (m)	ritmo (m)	ρυθμός (m)
8. triomphe (m)	triunfo (m)	trionfo (m)	θρίαμβος (m)

9. vent (m)	viento (m)	vénto (m)	άνεμος (m)<br>αέρος (m)
10. balcon (m)	balcón (m)	balcone (m)	ζεώστης (m)<br>μπαλκόνι (n)
11. succès (m)	éxito (m)	succèsso (m)	θρίαμβος (m)
12. sommeil (m)<br>rêve (m)<br>songe (m)	ueno (m)	sónno (m)<br>sógno (m)	ύπνος (m)
13. sens (m)	sentido (m)	sènso (m)	έννοια (f)<br>λόγος (m)

Table 2. Feminine gender universalities

ENGLISH	UKRAINIAN	RUSSIAN	POLISH	BULGARIAN	GERMAN
1.atmosphere	атмосфера (f)	атмосфера (f)	atmosfera (f)	атмосфера (f)	Atmosphäre (f)
2. drought	посуха (f)	засуха (f)	posucha (f)	суша (f)<br>засуха (f)	Dürre (f)
3. clinic	клініка (f)	клиника (f)	klinika (f)	клиника (f)	Klinik (f)
4. library	бібліотека (f)	библиотека (f)	biblioteka (f)	библиотека (f)	Bibliothek (f)
5. guitar	гітара (f)	гитара (f)	gitara (f)	китара (f)	Gitarre (f)
6. bomb	бомба (f)	бомба (f)	bomba (f)	бомба (f)	Bombe (f)
7. reality	дійсність (f)<br>реальність (f)	действитель ность (f)<br>реальность (f)	rzeczywistoś ć (f)	реалност (f)	Realität (f)<br>Wirklichkeit (f)
8.independen ce	незалежніст ь (f)	независимос ть (f)	niezależność (f)	независимос т (f)	Unabhängigk eit (f)
9. democracy	демократія (f)	демократия (f)	demokracja (f)	демокрация (f)	Demokratie (f)
10. fashion	мода (f)	мода (f)	moda (f)	мода (f)	Mode (f)
11. career	кар'єра (f)	карьера (f)	kariera (f)	кариера (f)	Karriere (f)<br>Laufbahn (f)
12.virtue	чеснота (f)	добродетель (f)	cnota (f)	добродетел (f)	Tugend (f)
13. friendship	дружба (f)	дружба (f)	przyjaźń (f)	дарба (f)<br>дружба (f)	Freundschaft (f)
14. responsibility	відповідаль ність (f)	ответственн ость (f)	odpowiedzial ność (f)	отговорност (f)	Verantwortun g (f)
15. discipline	дисципліна (f)	дисциплина (f)	dyscyplina (f)	дисциплина (f)	Disziplin (f)

16. emotion	емоція (f)	эмоция (f)	emocja (f)	емоция (f)	Emotion (f)
17. morals	мораль (f)	мораль (f)<br>нравственность (f)	moralność (f)	мораль (f)	Moral (f)
18. music	музика (f)	музыка (f)	muzyka (f)	музика (f)	Musik (f)
19. comedy	комедія (f)	комедия (f)	komedia (f)	комедия (f)	Komödie (f)
20. tragedy	трагедія (f)	трагедия (f)	tragedia (f)	трагедия (f)	Tragödie (f)
21. weakness	слабкість (f)	слабость (f)	słabość (f)	слабост (f)	Schwäche (f)
22. criticism	критика (f)	критика (f)	krytyka (f)	критика (f)	Kritik (f)
23. hostility	ворожнеча (f)	вражда (f)	wrogość (f)	вражда (f)	Feindschaft (f)
24. religion	релігія (f)	религия (f)	religia (f)	религия (f)	Religion (f)
25. harmony	гармонія (f)	гармония (f)	harmonia (f)	хармония (f)	Harmonie (f)
26.nature	природа (f)	природа (f)	przyroda (f)	природа (f)	Natur (f)
27.beauty	краса (f)	красота (f)	piękność (f)<br>vroda (f)	красота (f)<br>хубост (f)	Schönheit (f)
28. grace	грація (f)	грация (f)	gracja (f)	грация (f)	Grazie (f)<br>Anmut (f)
29. eternity	вічність (f)	вечность (f)	wieczność (f)	вечност (f)	Ewigkeit (f)
30. street	вулиця (f)	улица (f)	ulica (f)	улица (f)	Straße (f)
31. kitchen	кухня (f)	кухня (f)	kuchnia (f)	кухня (f)	Küche (f)
32. night	ніч (f)	ночь (f)	noc (f)	нощ (f)	Nacht (f)
33. rose	троянда (f)	роза (f)	róża (f)	роза (f)	Rose (f)
34. rage	лють (f)	ярость (f)	wściektość (f)	ярост (f)	Wut (f)
35. threat	погроза (f)	угроза (f)	groźba (f)	заплаха (f)<br>закана (f)	Drohung (f)
36. wound	рана (f)	рана (f)	rana (f)	рана (f)	Wunde (f)
37. bottle	пляшка (f)	бутылка (f)	butelka (f)	бутилка (f)	Flache (f)
38. irony	иронія (f)	ирония (f)	ironia (f)	ирония (f)	Ironie (f)
39. freedom	свобода (f)<br>воля (f)	свобода (f)	wolność (f)	свобода (f)	Freiheit (f)
40. energy	енергія (f)	энергия (f)	energia (f)	енергия (f)	Energie (f)<br>Tatkraft (f)
41. idea	ідея (f)	идея (f)	idea (f)	идея (f)	Idee (f)
42. poetry	поезія (f)	поэзия (f)	poezija (f)	поезия (f)	Dichtung (f)
43. aid<br>help	допомога (f)	помощь (f)	pomoc (f)	помощ (f)	Hilfe (f)
44.offense	образа (f)	обида (f)	krzywda (f)	обида (f)	Kränkung (f)
45. melody	мелодія (f)	мелодия (f)	melodia (f)	мелодия (f)	Melodie (f)

Table 2. (continuation)

FRENCH	SPANISH	ITALIAN	GREEK
1.atmosphère (f)	atmósfera (f)	atmosfera (f)	ατμόσφαιρα (f)
2.sécheresse (f)	sequia (f)	siccità (f)	ξπρασία (f)
3.clinique (f)	clinica (f)	clinica (f)	κλινική (f)
4.bibliothèque (f)	biblioteca (f)	biblioteca (f)	βιβλιοθήκη (f)
5.guitare (f)	guitarra (f)	chitarra (f)	κιθάρα (f)
6.bombe (f)	bomba (f)	bomba (f)	βόμβα (f)
7.réalité (f)	realidad (f)	realtà (f)	πραγματι-κότητα {-ης} (f)
8.indépendance (f)	independencia (f)	indipendenza (f)	ανεξαρτησία (f)
9.démocratie (f)	democracia (f)	democrazia (f)	δημοκρατία (f)
10.mode (f)	moda (f)	moda (f)	μόδα (f)
11.carrière (f)	carrera (f)	carriera (f)	σταδιοδρομία (f)<br>καριέρα (f)
12.vertu (f)	virtud (f)	virtú (f)	αρετή (f)
13.amitié (f)	amistad (f)	amicizia (f)	φιλία (f)
14.responsabilité (f)	responsabildad (f)	responsabilità (f)	ευθύνη (f)
15.discipline (f)	disciplina (f)	disciplina (f)	πειθαρ χία (f)
16. emotion (f)	emoción (f)	emozione (f)	συγκίνηση (f)
17. morale (f)	moral (f)	morale (f)	ήθική (f)
18.musique (f)	músico (f)	musica (f)	μουσική (f)
19.comédie (f)	comedia (f)	commedia (f)	κωμωδία (f)
20.tragédie (f)	tragedia (f)	tragedia (f)	τραγωδία (f)
21.faiblesse (f)	debilidad (f)	debolezza (f)	αδυναμία (f)
22.critique (f)	critica (f)	critica (f)	κριτική (f)
23.animosité (f)	enemistad (f)	inimicizia (f)	εχθρότητα (f)
24. religion (f)	religión (f)	religione (f)	θρησκεία (f)
25. harmonie (f)	armonia (f)	armonia (f)	άρμονια (f)
26. nature (f)	naturaleza (f)	natura (f)	φύση (f)
27. beauté (f)	belleza (f)	bellézza (f)	ομορφιά (f)<br>κάλλος (n)
28. grâce (f)	gracia (f)	grazia (f)	χάρη {-ις} (f)

			κομψότητα (f)
29. éternité (f)	eternidad (f)	eternità (f)	αιωνιότητα (f)
30. rue (f)	calle (f)	via (f)<br>strada (f)	οδός (f)<br>δρόμος (m)
31. cuisine (f)	cuisine (f)	cucina (f)	κουζίνα (f)<br>μαγειρείο (n)
32. nuit (f)	noche (f)	notte (f)	νύχτά (f)
33. rose (f)	rosa (f)	rŏʃa (f)	τριαντάφυλλο (n)<br>τριανταφυλλιά (f)
34. fureur (f)	furia (f)	furia (f)	λύσσα (f)
35. menace (f)	amenaza (f)	minaccia (f)	απειλή (f)
36. plaie (f)	herida (f)	ferita (f)	τραύμα (n)<br>πληγή (f)
37. bouteille (f)	botella (f)	bottiglia (f)<br>pet (m)	φιάλητ (f)<br>μπουκάλι (n)
38. ironie (f)	ironia (f)	ironia (f)	ειρωνεία (f)
39. liberte (f)	libertad (f)	libertà (f)	ελευθερία (f)
40. idée (f)	idea (f)	idea (f)	ιδέα (f)
41. poésie (f)	poesia (f)	poesia (f)	ποίηση (f)
42. aide (f)	ayuda (f)	aiuto (f)<br>assistenza (f)	βοήθεια (f)
43.vexation (f)<br>offense (f)	ofenza (f)	offésa (f)	προσβολή (f)
44. melodie (f)	melodia (f)	melodia (f)	μελωδία (f)
45.vexation (f)<br>offense (f)	ofenza (f)	offésa (f)	προσβολή (f)

APPENDIX B

Absolute grammatical gender universalities of alternative type

* In tables 1, 2 of this appendix, alternative variants are set in bold

Table 1. Masculine gender universalities:

ENGLISH	UKRAINIAN	RUSSIAN	POLISH	BULGARIAN	GERMAN
1. stream	потік (m)	поток (m)	potok (m)	поток (m)<br>ручей (m)	Strom (m)
2. hill	пагорб (m)	холм (m)	**wzgórze (n)**	хълм (m)	Hügel (m)

			**wzgórek (m)**	ряд (m)	
3. park	парк (m)	парк (m)	park (m)<br>ogród (m)	**парк (m)**<br>**градина (f)**	Park (m)
4. diamond	діамант (m)	алмаз (m)	diament (m)	диамант (m)<br>елмаз (m)	Diamant (m)
5. temple	храм (m)	храм (m)	**świątynia (f)**<br>**kościół (m)**	храм (m)	Tempel (m)
6. port	порт (m)	порт (m)	port (m)	пристанище (m)	Hafen (m)
7. motor	мотор (m)	мотор (m)	motor (m)	мотор (m)	Motor (m)
8.computer	комп'ютер (m)	компьютер (m)	komputer (m)	компютър (m)	Computer (m)
9. character	**характер (m)**<br>**вдача (f)**	нрав (m)<br>характер (m)	character (m)	нрав (m)<br>характер (m)	Charakter (m)
10. style	стиль (m)	стиль (m)	styl (m)	стил (m)	Stil (m)
11. myth	міф (m)	миф (m)	mit (m)	**мит (m)**<br>**измислица (f)**	**Mythus (m)**<br>**Sage (f)**
12.terror	жах (m)	ужас (m)	przerażenie (n)	ужас (m)	**Grauen (n)**<br>**Schrecken (m)**
13. hurricane	буревій (m)	ураган (m)	huragan (m)<br>orkan (m)	ураган (m)	Orkan (m)

Table 1. (continuation)

FRENCH	SPANISH	ITALIAN	GREEK
1. torrent (m)	torrente (m)	torrènte (m)	**χείμαρρος (m)**<br>**ρέμα (n)**<br>**ποτάμι (n)**
2. **mamelon (m)**<br>**coteau (m)**<br>**colline (f)**	**colina (f)**<br>**cerra (m)**	**collina (f)**<br>**pòggio (m)**	λόφος (m)
3. pare (m)	parque (m)	parco (m)	**κήπος (m)**<br>**πάρκο (n)**
4. diamant (m)	diamante (m)	diamante (m)	**διαμάντι (n)**<br>**ἀδάμας (m)**
5. temple (m)	templo (m)	tempio (m)	**ναός (m)**<br>**ιερό (f)**

			**τέμενος (n)**
6. port (m)	puerto (m)	porto (m)	**λιμάνι (n)**<br>**λιμήν (m)**
7. moteur (m)	motor (m)	motore (m)	**μοτέρ (n)**<br>**κινητήρας (m)**
8. ordinateur (m)<br>computer (m)	**computador (m)**<br>**computadora (f)**	computer (m)	υπολογιοτής (m)
9. caractère (m)<br>naturel (m)	carácte (m)	caràttere (m)	χαρακτήρας (m)
10. style (m)	estilo (m)	stile (m)	**στυλ (n)**<br>**ρυθμός (m)**<br>**ὑφος (n)**
11. mythe (m)	mito (m)	mito (m)	μύθος (m)
12. effroi (m)	horror (m)	terróre (m)	**τρόμος (m)**<br>**φρίκη (f)**
13. ouragan (m)	huracan (m)	uragano (m)	**σίφουνας (m)**<br>**λαίλαπα (f)**

Table 2. Feminine gender universalities

ENGLISH	UKRAINIAN	RUSSIAN	POLISH	BULGARIAN	GERMAN
1. plain	рівнина (f)	равнина (f)	równina (f)	равнина (f)	**Ebene (f)**<br>**Flachland (n)**
2. cave	печера (f)	пещера (f)	jaskinia (f)<br>grota (f)	печера (f)	Höhle (f)
3. moisture	волога (f)	влага (f)	wilgoć (f)	влага (f)<br>течност (f)<br>вода (f)	Feuchtigkeit (f)
4. midnight	північ (f)	полночь (f)	północ (f)	полунощ (f)<br>среднощ (f)	Mitternacht (f)
5. lamp	лампа (f)	лампа (f)	lampa (f)	лампа (f)	Lampe (f)
6. joy	радість (f)<br>втіха (f)	радость (f)	radość (f)	радост (f)	Freude (f)
7. soul	душа (f)	душа (f)	dusza (f)	душа (f)	Seele (f)
8. hope	надія (f)	надежда (f)	nadzieja (f)	надежда (f)	Hoffnung (f)
9. strength<br>force	сила (f) /<br>снага (f)	сила (f)<br>энергия (f)	sila (f)	сила (f)<br>енергия (f)	Kraft (f)<br>Tatkraft (f)
10.	реклама (f)	реклама (f)	reklama (f)	реклама (f)	Reklame (f)

advertising					
11. truth	правда (f)	правда (f)	prawda (f)	истина (f)	Wahrheit (f)
12.wisdom	мудрість (f)	мудрость (f)	mądrość (f)	мъдрост (f)	Weisheit (f)
13.defense	захист (m)<br>оборона (f)	защита (f)<br>оборона (f)	obrona (f)	защита (f)<br>отбрана (f)	**Schutz (m)**<br>**Verteidigung (f)**<br>**Abwehr (f)**
14. disease illness	хвороба (f)	болезнь (f)	choroba (f)	болест (f)	Krankheit (f)
15. slander	**наклеп (m)**<br>**обмова (f)**	**клевета (f)**<br>**оговор (m)**	obmowa (f)	клевета (f)	Verleumdung (f)
16. culture	культура (f)	культура (f)	kultura (f)	култура (f)	Kultur (f)
17. revenge	помста (f)	месть (f)	zemsta (f)	мъст (f)<br>отмъщение (n)	Rache (f)
18. gratitude	подяка (f)	благодарность (f)	**wdzięczność (f)**<br>**podziękowanie (n)**	благодарност (f)<br>признателност (f)	**Dankbarkeit (f)**<br>**Dank (m)**

Table 2. (continuation)

FRENCH	SPANISH	ITALIAN	GREEK
1.plaine (f)	llanura(f)	pianura (f)	πεδιάδα(f)
2. caverne (f)<br>grotte (f)	cueva (f)<br>caverna (f)<br>gruta (f)	caverna (f)<br>grotta(f)<br>spelonca(f)	**σπήλαιο (n)**<br>**όχηλιά (f)**
3. humidite (f)	humedad (f)	**umidézza (f)**<br>**ùmido (m)**	υγρασία (f)
4. **minuit (m)** (f)	medianoche (f)	ezzanotte (f)	μεσάνυχτα(f)
5. lampe (f)	lampara (f)	lampada (f)<br>valvula (f)	**λάμπα (f)**<br>**λαμπατήρας (m)**
6. joie (f)	**alegria (f)**<br>**gozo (m)**	**giòia (f)**<br>**giúbilo (m)**	μεάλη χαρα (f)
7.âme (f)	alma (f)	**ànima (f)**<br>**animo (m)**	ψυχή (f)
8. espoir (m)<br>espérance (f)	esperanza (f)	speranza (f)	ελπίδα (f)

9.force (f) /<br>vigueur (f)	**fuerza (f) /<br>vigor (m)**	**fórza (f) /<br>vigòre (m)**	**δύναμη (f) /<br>σφρίγος (m)**
10. réclame (f)<br>publicité (f)	**publicidad (f)<br>anuncio (m)**	pubblicità (f)<br>reclame (f)	ρεκλάμα (f)<br>διαφήμιση (f)
11. vérité (f)	verdad (f)	**verità (f)<br>véro (m)**	αλήθεια (f)
12. sagesse (f)	sabiduria (f)	**saggézza (f)<br>sénno (m)**	σοφία (f)
13. défense (f)	defensa (f)	difesa (f)	άμυνα (f)<br>προστασία (f)
14. **maladie (f)<br>mal (m)**	enfermedad (f)	**malattía (f)<br>male (m)**	ασθένεια (f)
15.calomnie (f)<br>dénonciation (f)	calumnia (f)	calúnnia (f)	ουχοφαγτία (f)<br>διαβολή (f)
16. culture (f)	cultura (f)	cultura (f)	**πολττσμός (m)<br>κουλτούρα(f)**
17. vengeance (f)	venganza (f)	vendetta (f)	εκδίκηση (f)
18.reconnaissance (f)<br>gratitude (f)	**agradecimiento (m)<br>gratitud (f)**	gratitúdine (f)	ευγνωμοσύνη (f)

APPENDIX C

Restricted grammatical gender universalities

* In tables 1, 2 of this appendix, the variants deviating from the dominant gender tendency are set in bold

Table 1. Masculine gender universalities

ENGLISH	UKRAINIAN	RUSSIAN	POLISH	BULGARIAN	GERMAN
1. palace	палац (m)	дворец (m)	pałac (m)	дворец (m)	Palast (m)
2. van	фургон (m)	фургон (m)	furgon (m)	фургон (m)	Van (m)
3. bus	автобус (m)	автобус (m)	autobus (m)	автобус (m)	Autobus (m)
4. helicopter	вертоліт (m)<br>гелікоптер (m)	вертолёт (m)	helikopter (m)	вертолет (m)<br>хеликоптер (m)	Hubschraube r (m)
5. tank	танк (m)	танк (m)	czołg (m)<br>tank (m)	танк (m)	Panzer (m)<br>Tank (m)
6.(tele)phone	телефон (m)	телефон (m)	telefon (m)	телефон (m)	Telefon (n)<br>Fernsprecher (m)

7. laser	лазер (m)	лазер (m)	laser (m)	лазер (m)	Laser (m)
8. carpet	килим (m)	ковёр (m)	dywan (m)<br>kobierzec (m)	килим (m)	Teppich (m)
9. pencil	олівець (m)	карандаш (m)	ołówek (m)	молив (m)	Bleistift (m)
10. sweater	світер (m)	свитер (m)	sweter (m)	полувер (m)	Rollkragenpullover (m)
11. oxygen	кисень (m)	кислород (m)	tlen (m)	кислород (m)	Sauerstoff (m)
12. gene	ген (m)	ген (m)	gen (m)	ген (m)	Gen (m)
13. nerve	нерв (m)	нерв (m)	nerw (m)	нерв (m)	Nerv (m)
14. crystal	кришталь (m)	хрусталь (m)	kryształ (m)	кристал (m)	Kristall (m)
15. end	кінець (m)<br>край (m)	конец (m)<br>исход (m)	koniec (m)<br>wynik (m)	край (m)	Ausgang (m)<br>Schluß (m)<br>Ende (n)
16. humor	гумор (m)	юмор (m)	humor (m)	хумор (m)	Humor (m)
17. rice	рис (m)	рис (m)	ryż (m)	ориз (m)	Reis (m)
18. apparatus	апарат (m)	аппарат (m)	aparat (m)	апарат (m)<br>уред (m)	Apparat (m)<br>Vorrichtung (f)
19. tv set	телевізор (m)	телевизор (m)	telewizor (m)	телевизор (m)	Fernsehempfänger (m)<br>Fernseher (m)
20. tap / faucet	кран (m)	кран (m)	kran (m)	кран (m)	Wasserhahn (m)
21. day	день (m)<br>днина (f)	день (m)	dzień (m)	ден (m)	Tag (m)
22. thunder	грім (m)	гром (m)	grzmot (m)<br>grom (m)	гръмеж (m)<br>гръмотевица (f)	Donner (m)
23. wood	ліс (m)	лес (m)	las (m)<br>bór (m)	**гора (f)**	Wald (m)
24. garden	сад (m)	сад (m)	ogród (m)	**градина (f)**	Garten (m)
25. trunk	стовбур (m)	ствол (m)	pień (m)	**стебло (n)**	Stamm (m)
26. airport	аеропорт (m)<br>летовище (n)	аэропорт (m)	**lotnisko (n)**	аеродрум(m)<br>аерогара (f)<br>летище (n)	Flughafen (m)
27. satellite	спутник (m)	спутник (m)	**satelita (f)**	спътник (m)	Satellit (m)
28. world	світ (m)	мир (m)	świat (m)	свят (m)	**Welt (f)**

29. paradise	рай (m)	рай (m)	raj (m)	рай (m)	**Paradies (n)**

Table 1.(continuation)

FRENCH	SPANISH	ITALIAN	GREEK
1. palais (m)	palacio (m)	palazzo (m)	**παλάτι (n)**<br>**μέγαρο (n)**<br>ανάκτορα (pl)
2. fourgon (m)	furgón (m)<br>furgoneta (f)	furgone (m)	**φορτηγό (n)**
3. autobus (m)	autobús (m)	autobus (m)<br>autopullman (m)	**λεωφορείο (n)**
4. hélicoptère (m)	helicóptero (m)	elicottero (m)	**ελικόπτερο (n)**
5. char (m)<br>tank (m)	tanque (m)	carro (m) armato	**τανκ (n)**
6. téléphone (m)	teléfono (m)	telefono (m)<br>apparecchio (m)	**τηλέφωνο (n)**
7. laser (m)	làser (m)	laser (m)	**λέιζερ (n)**
8. tapis (m)	tapiz (m)<br>alfombra (f)	tappeto (m)	**χαλί (n)**<br>**τάπης (m)**
9. crayon (m)	làpiz (m)<br>lapicero (m)	lapis (m)<br>matita (f)	**μολύβι (n)**
10. sweater (m)<br>chandail (m)	jersey (m)<br>suéter (m)	maglione (m)	**πουλόβερ (n)**
11. oxygène (m)	oxígeno (m)	ossigeno (m)	**ὀξυγόνο (n)**
12. gène (m)	gene (m)	gene (m)	**γονίδιο (n)**
13. nerf (m)	nervio (m)	nervo (m)	**νεῦρο (n)**
14. cristal (m)	cristal (m)	cristallo (m)	**κρύσταλλο (n)**
15. fin (f)<br>terme (m)	fin (m)<br>final (m)<br>extremo (m)	fine (f)<br>termine (m)	**τέλος (n)**<br>**πέρας (n)**
16. humour (m)	humor (m)	umorismo (m)	**χιούμορ (n)**
17. riz (m)	arroz (m)	riso (m)	**ρύζι (n)**<br>**δρυζα (f)**
18. appareil (m)	aparato (m)	apparecchio (m)	**συσκευή (f)**<br>**μηχάνημα (n)**

19. téléviseur (m)	televisor (m)	telewizor (m)	**τιμή (f)**
20. robinet (m)	llave (f)<br>grifo (m)	rubinetto (m)	**βρύστη (f)**
21. jour (m)	dia (m)	giorno (m)<br>giornata (f)	**μέρα(f)**
22. tonnere (m)	trueno (m)	tuòno (m)	**βροντή (f)**
23. bois (m)<br>forêt (f)	bosque (m)	bòsco (m)<br>forèsta (f)	δάσος (n)<br>λχόγγος (m)
24. jardin (m)<br>verger (m)	jardin (m)<br>huerto (m)	giardino (m)	κήπος(m)
25. trone (m)	tronco (m)	trónco (m)	κορμός (m)
26. aéroport (m)	aeropuerto (m)	aeroporto (m)<br>aerodromo (m)	άεροδρόμιο (n)<br>άερολιμένος (m)
27. satellite (m)	satélite (m)	satellite (m)<br>sputnik (m)	σπούτνικ (m)
28. monde (m)	mundo (m)	móndo (m)	κόσμος (m)
29. paradis (m)	paraiso (m)<br>edén (m)	paradiso (m)	παράδειδος (m)

Table 2. Feminine gender universalities

ENGLISH	UKRAINIAN	RUSSIAN	POLISH	BULGARIAN	GERMAN
1. drop	крапля (f)	капля (f)	kropla (f)	капка (f)	**Tropfen (m)**
2. skirt	спідниця (f)	юбка (f)	spódnica (f)	пола (f)	**Rock (m)**
3. faith	віра (f)	вера (f)	wiara (f)	увереност (f)<br>доверие (n)	**Glaube(n) (m)**
4. glory	слава (f)	слава (f)	sława (f)	слава (f)	**Ruhm (m)**
5. envy	заздрість (f)	зависть (f)	zawiść (f)	завист (f)	**Neid (m)**
6. land	суша (f)<br>суходіл (m)	суша (f)	ziemia (f)	земя (f)	**Land (n)**
7. prayer	молитва (f)	молитва (f)	modlitwa (f)	молитва (f)	**Gebet (n)**
8. galaxy	галактика (f)	галактика (f)	galaktyka (f)	галактика (f)	Galaxie (f)
9. earth<br>soil	земля (f)	земля (f)	ziemia (f)	земя (f)	Erde (f)
10. passion	пристрасть (f)	страсть (f)	namięność (f)	страст (f)	Leidenschaft (f)
11. tear	сльоза (f)	слеза (f)	łza (f)	сълза (f)	Träne (f)

12. humanity	людяність (f)	человечность (f)	ludzkość (f)<br>człowieczeństwo (n)	човечество (n)	Menschlichkeit (f)<br>Humanitat (f)
13. anxiety	тривога (f)	тревога (f)	trwoga (f)	**безпокойство (n)**	Unruhe (f)<br>Aufregung (f)
14. guilt	провина (f)	вина (f)	wina (f)	**вина (f)**	Schuld (f)<br>Vergehen (n)
15. cradle	колиска (f)	колыбель (f)	kolebka (f)	люлка (f)	Wiege (f)
16. fatigue	втома (f)	усталость (f)	**zmęczenie (n)**	умора (f)	Müdigkeit (f)

Table 2. (continuation)

FRENCH	SPANISH	ITALIAN	GREEK
1. goutte (f)	gota (f)	gòccia (f)	σταγόνα (f)
2. jupe (f)	falda (f)<br>saya (f)	gonna (f)	φούκοστα (f)
3. foi (f)	fe (f)	féde (f)	πίστη (f)<br>εμπιοτοσύνη (f)
4. gloire (f)	gloria (f)	glòria (f)	φήμη (f)<br>δόξα (f)
5. envie (f)	envidia (f)	invidia (f)	ζήλια (f)
6. terre (f)	tierra (f)	tèrra (f)	ξηρά (f)<br>στεριά (f)
7. prière (f)	oraciòn (f)	preghiera (f)	προσευχή (f)
8. galaxie (f)	galaxia (f)	constellazióne (f)	**γαλαξίας (m)**<br>**αχερόδρομος (m)**
9. terre(f)	tierra(f)	tèrra(f)	**χώμα(n)**
10. passion (f)	pasión (f)	passione (f)<br>adore (m)	**πάθος (n)**
11. larme (f)	lagrima (f)	làcrima (f)	**δάκρυ (n)**
12. humanité (f)	humanidad (f)	umanità (f)	ανθρωπιά (f)
13. anxiété (f)<br>angoisse (f)	alarma (f)<br>inquietud (f)	angoscia (f)<br>ansia (f)	ανησυχία (f)<br>ταραχή (f)

14. faute (m)<br>tort (m)	culpa (f)	cólpa (f)	ενοχή (f)
15. **berceau (m)**	cuna (f)	culla (f)	κούνια (f)
16. fatigue (f)	cansancio (m)<br>fatiga (f)	stanchezza (f)	κούραση (f)

APPENDIX D

Table 1. Non-feminine grammatical gender universalities

ENGLISH	UKRAINIAN	RUSSIAN	POLISH	BULGARIAN	GERMAN
1. talent	талант (m)	талант (m)	talent (m)	талант (m)	Talent (n)
2. feeling	почуття (n)	чувство (n)	czucie (n)<br>uczucie (n)	чувство (n)<br>усещане (n)<br>усет (m)	Sinn (m)
3. miracle	диво (n)	чудо (n)	cud (m)<br>cudo (n)<br>dziw (m)	чудо (n)	Wunder (n)
4. evil	зло (n)	зло (n)	zło (n)	зло (n)	Übel (n)<br>Böse (n)
5. theatre	театр (m)	театр (m)	teatr (m)	театър (m)	Theater (n)
6. museum	музей (m)	музей (m)	museum (n)	музей (m)	Museum (n)
7. cemetery	кладовище (n)<br>цвинтар (m)	кладбище (n)	cmentarz (m)	гробища (pl)	Friedhof (m)
8. aircraft	літак (m)	самолёт (m)	samolot (m)	самолет (m)	Flugzeug (n)
9. radar	радар (m)	радар (m)	radar (m)	радар (m)	Radar (m), (n)
10. instrument	інструмент (m)	инструмент (m)	instrument (m)	инструмент (m)	Instrument (n)<br>Werkzeug (n)
11. cabinet	кабінет (m)	кабинет (m)	gabinet (m)	кабинет (m)	Arbeitszimmer (n)<br>Kabinett (n)
12. knife	ніж (m)	нож (m)	nóż (m)	нож (m)	Messer (n)
13. piano	піаніно (n)	пианино (n)	fortepian (m)	пиано (n)	Klavier (n)
14. drum	барабан (m)	барабан (m)	bęben (m)	барабан (m)<br>тъпан (m)	Trommel (f)
15. diary	щоденник (m)	дневник (m)	pamiętnik (m)	дневник (m)	Tagebuch (n)

			dziennik (m)		
16. document	документ (m)	документ (m)	dokument (m)	документ (m)	Dokument (n)
17. coat	пальто (n)	пальто (n)	płaszcz (m)<br>palto (n)	палто (n)	Mantel (m)
18. sky	небо (n)	небо (n)	niebo(n)	небо (n)	Himmel (m)
19. climate	клімат (m)	климат (m)	klimat (m)	климат (m)	Klima (n)
20. earthquake	землетрус (m)	землетрясение (n)	trzęsienie (n) ziemi	земетресение (n)<br>земетръс (m)	Erdbeben (n)
21.grain	зерно (n)	зерно (n)	ziarno (n)	зърно (n)<br>зрънце (n)	Korn (n)
22. mineral	мінерал (m)	минерал (m)	minerał (m)	минерален (m)	Mineral (n)
23. gold	золото (n)	золото (n)	złoto (n)	злато (n)	Gold (n)
24. iron	залізо (n)	железо (n)	żelazo (n)	желязо (n)	Eisen (n)
25. marble	мармур (m)	мрамор (m)	marmur (m)	мрамор (m)	Marmor (m)
26. baby	немовля (n)	младенец (m)	nemowlę (n)	бебе (n)	Säugling (m)
27. afternoon	полудень (m)	полдень (m)	połudmie (n)	пладне (n)	Mittag (m)
28.casino	казіно (n)	казино (n)	kasyno (n)	казино (n)	Kasino (n)
29. right	право (n)	право (n)	prawo (n)	право (n)	Recht (n)
30. crime	злочин (m)	преступление (n)	przestępstwo (n)<br>występek (m)	престъпление (n)	Verbrechen (n)<br>Frevel (m)
31. costume/suit	костюм (m)	костюм (m)	garnitur (m)<br>kostum (m)<br>stroj (m)	костюм (m)	Anzug (m)<br>Kostüm (n)

Table 1 (continuation)

FRENCH	SPANISH	ITALIAN	GREEK
1. talent (m)	talento (m)	talènto (m)	ταλέντο (n)
2. sens (m)<br>sentiment (m)	sentido (m)<br>sentimiento (m)	sènso (m)	αίσθημα (n)
3. miracle (m)<br>prodige (m)	milagro (m)<br>prodigio (m)	miràcolo (m)	θαύμα (n)<br>θαυμάσιο (n)
4. mal (m)	mal (m)	male (m)	κακό (n)
5. théâtre (m)	teatro (m)	teatro (m)	θέατρο (n)
6. musée (m)	museo (m)	museo (m)	μουσείο (n)

7. cimetière (m)	cementerio (m)<br>camposanto (m)	cimitèro (m)	νεκροταφείο (n)<br>κοιμητήριο (n)
8. avion (m)	avión (m)	aereo (m)<br>apparecchio (m)	αέροπλάνο (n)
9. radar (m)	radar (m)	radar (m)	ραντάρ (n)
10. instrument (m)<br>ustensile (m)<br>outil (m)	instrumento (m)<br>herramienta (f)<br>ustensilio (m)<br>util (m)	strumento (m)<br>ustensile (m)<br>attrezzo (m)	εργαλείο (n)
11. cabinet (m)	gabinete (m)<br>despacho (m)	studio (m)<br>gabinetto (m)	γραφείο (n)<br>ἰατρεῖο{ν (n)
12. couteau (m)	cuchillo (m)	coltello (m)	μαχαίρι (n)<br>μάχαιρα (f)
13. piano (m)	piano (m)	piano (m)	πιάνο (n)
14. tambour (m)	tambor (m)	tamburo (m)	τύμπανο (n)
15. journal (m)	diario (m)	diario (m)	ημερολόγιο (n)
16. document (m)<br>pièce (f)	documento (m)	documento (m)<br>certificato (m)	έγγραφο (n)
17. pardessus (m)<br>manteau (m)	abrigo (m)	cappotto (m)<br>paltò (m)	παλτό (n)
18. ciel (m)	cielo (m)	cièlo (m)	ουρανός (m)
19. climat (m)	clima (m)	clima (m)	κλίμα (n)
20. tremblement (m) de terre; séisme (m)	terremoto (m)<br>sismo (m)	terremoto (m)<br>seismo (m)	σεισμός (m)
21. grain (m)	grano (m)<br>granado (m)	chicco (m)<br>grano (m)	κόκκος (m)
22. minéral (m)	mineral (m)	minerale (m)	ορυκτό (n)
23. or (m)	ora (m)	oro (m)	χρυσός (m)<br>χρυσάφι(n)
24. fer (m)	hierro (m)	ferro (m)	σίδερο (n)<br>σίδηρος (m)
25. marbre (m)	mármol (m)	marmo (m)	μάρμαρο (n)
26. bébé (m)<br>nourrisson (m)<br>poupon (m)	niño de pecho (m)	poppante (m)	μωπό (n)
27. midi (m)	mediodia (m)	mezzogiorno (m)	μεσημέρι(n)

28.casino (m)	casino (m)	casino (m)	καζίνο (n)
29. droit (m)	derecho (m)	diritto (m)	δικαίωμα (n)
30. crime (m)<br>délit (m)	delito (m)<br>crimen (m)	delitto (m)	έγκλημα (n)<br>αδίκμα (n)
31. costume (m)	traje (m)	costume (m)	κοστούμι (n)<br>ταγιέρ (n)
